# Efficacy of Postoperative Radiographs After Intramedullary Nailing of the Tibia and Femur: When Are They Useful?

**DOI:** 10.5435/JAAOSGlobal-D-23-00069

**Published:** 2023-06-06

**Authors:** Vivek Nair, Jennifer Lewis, Miguel Daccarett, Douglas Dirschl, Kelly Hynes, Jason Strelzow

**Affiliations:** From the University of Chicago Pritzker School of Medicine, Chicago, IL (Mr. Nair), and the UChicago Medicine Department of Orthopaedic Surgery and Rehabilitation Medicine, Chicago, IL (Ms. Lewis, Dr. Daccarett, Dr. Dirschl, Dr. Hynes, and Dr. Strelzow).

## Abstract

Postoperative radiographs are used to monitor fractures of the tibia and femur after intramedullary fixation. This study sought to examine how frequently these radiographs change management.

**Methods:**

This was a single-center chart review of patients over a 4-year period at a level I trauma center. Radiographs were defined as either performed for routine surveillance or performed with some clinical correlate on history and examination. Participants received intramedullary nailing for diaphyseal fractures of the femur or tibia. Patients required at least one postoperative radiograph. All patients were subject to our institution's follow-up protocol: visits at 2, 6, 12, and 24 weeks. Radiographs that changed management were those that led to alterations in follow-up, directed counseling, or contributed to the decision to proceed with revision surgery.

**Results:**

A total of 374 patients were found. Two hundred seventy-seven received at least one post-op radiograph. The median follow-up was 23 weeks. Six hundred seventeen total radiographs were reviewed. Nine radiographs contributed to a change in management (9/617 = 1.5%). No surveillance radiograph taken before 14 weeks resulted in changes in management.

**Discussion:**

Our results suggest that radiographs taken in the first 3 months post-op in asymptomatic patients treated with lower extremity intramedullary rods do not result in changes to clinical management.

Radiographs taken during postoperative clinic visits are often used to surveil for complications of diaphyseal fractures of the femur and tibia treated with intramedullary nailing.^1,2^ The goal of this surveillance is to monitor for appropriate healing and assess for complications, including those requiring revision surgery.^3^ Among complications, nonunion is of particular note because of patient morbidity and the notable cost of intervention.^4,5,6^ As such, history, physical examination, and regular postoperative surveillance radiographs are common and central facets of fracture care to monitor for postfixation complications.^7,8^

The utility of routine imaging studies for surveillance in asymptomatic patients with long bone fractures is currently unclear. One survey of orthopaedic surgeons performing long bone fixation found that most respondents currently were obtaining surveillance radiographs at 2 weeks postoperatively. However, about a quarter of those surgeons were open to forgoing early surveillance radiographs under the belief that such studies are often of low diagnostic yield.^9^ A subsequent trial by the same group randomized patients with stable fractures of the femur, tibia, humeral shaft, ankle, and clavicle to scheduled postoperative surveillance radiographs versus only receiving imaging when the surgeon documented some clinical concern warranting the imaging.^10^ The study found that scheduled surveillance radiographs did not discover more complications than what would be detected by clinical indications alone. Another study investigating early imaging of distal radius fractures found that patients who underwent early surveillance imaging had their management change in only 4% of cases.^11^ Work by Bohl et al has demonstrated the low utility of an AP radiograph in addition to a lateral view following anterior cervical decompression and fusion procedures, as well as the fact that only the very initial postoperative radiograph after distal radius fracture fixation was of notable value.^12,13^ However, there has been no analogous work in determining the utility of surveillance imaging in diaphyseal fractures of the tibia and femur and unnecessary cost in sequential postoperative imaging of distal radius fractures.

This investigation sought to examine how frequently postoperative radiographs change management for patients treated with intramedullary rods for diaphyseal fractures of the femur and tibia. We grouped these fractures together because at our institution they tend to occur in similar patient cohorts, in the setting of similar mechanisms of injury, and are subject to the same postoperative follow-up protocol. The study hypothesis was that early surveillance radiographs obtained in the first 6 weeks postoperatively without a clinically directed indication do not change patient management. We also sought to conduct a cost analysis and determine the impact and implications of decreased postoperative surveillance.

## Methods

This was a single-center retrospective study. A detailed chart review through the hospital's electronic health record was performed. Patients were identified if they had long bone injuries of the femur or tibia (defined as Orthopaedic Trauma Association (OTA) 32 and 42 fractures) and were treated with intramedullary nailing at our academic level I trauma center from 2016 through 2020. For inclusion in the analysis, patients required at least one postoperative radiograph and clinical follow-up visit. Patient demographics recorded included age at the time of injury, sex, height, weight, body mass index, interval between injury and definitive fixation, presence of fracture comminution, open versus closed fracture status, and number of interlocking screws used during the fixation. All patients were skeletally mature at the time of injury.

The authors performed a retrospective chart review including manual review of all clinical assessments and plans and radiographs taken during each visit. Indications for radiograph orders were reviewed, and radiographs were divided into surveillance radiographs and clinically indicated radiographs. Surveillance radiographs were defined as those radiographs obtained as part of a general practice pattern for postoperative patients without a documented historical or clinically focused concern. Clinically indicated radiographs were defined as any radiograph in which there was a documented concern from the patient or care team (ie, patient had pain, prominent metalwork was observed, or symptomatic nonunion was clinically present). In addition, imaging and chart review was then evaluated for the presence of any change in management. All images were also compared with intraoperative images as a baseline to detect unexpected findings not documented in the clinical or radiology documentation.

At the institution of this study, most patients with diaphyseal fractures of the femur and tibia are seen in the clinic at postoperative weeks 2, 6, 12, and 24. Postoperative imaging is taken at weeks 6, 12, and 24 or until fracture union is achieved. Changes in patient management were classified as alterations in patient follow-up schedule, patient-directed counseling on the need for symptom surveillance (pain at the delayed union site), nonsurgical treatments for suspected nonunion (ie, bone stimulator prescription and platelet-rich plasma injections), and the decision to proceed with revision surgery (ie, prominent metalwork removal or exchange nailing). Differences in the number of radiographs that change management between the asymptomatic screening radiographs and the clinically driven radiographs were analyzed using a chi-squared test, alpha = 0.05. To account for possible misclassification of management-altering screening radiographs, we performed a stepwise sensitivity analysis in which we repeated our tests of significance with sequential assumptions of misclassified radiographs until statistical significance was lost. All data analyses were conducted using Python, version 3.7.6.

We also sought to evaluate the cost associated with surveillance imaging after tibia and femur intramedullary fixation. As a part of this analysis, we used a time threshold of 12 weeks post-op. This timing both intersects with our institution's practice pattern and marks the time in which orthopaedic surgeons become more reliably able to predict nonunion based on patient symptomology and imaging findings.^14^ To quantify the cost associated with potentially unnecessary surveillance radiographs, we performed a cost analysis isolated strictly to the associated financial cost of performing the imaging. This excludes associated clinical costs, patient wait times, and societal costs associated with unnecessary radiation dosing. Costs of imaging studies at our institution are divided and have two prices: a “List Price,” which refers to the price quoted to the patient's insurance provider, and a “Research Price,” which reflects the lowest possible cost to cover the expense of performing the study. For a two-view radiograph of the tibia, the List Price is $1042.95, and the Research Price is $112.08. For a two-view radiograph of the femur, the List Price is $837.82, and the Research Price is $113.45.

## Results

**Figure 1:**
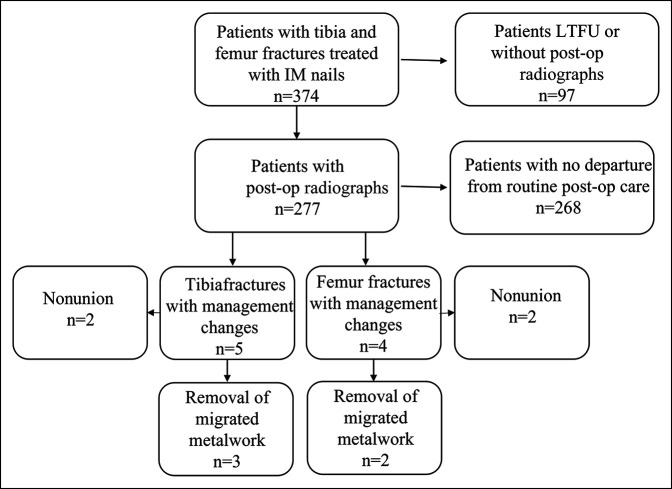
Flowsheet of cohort outcomes. IM = intramedullary, LTFU = lost to follow-up

Three hundred seventy-four patients who met the requisite inclusion criteria were discovered through chart review. Of these patients, 277 received at least one surveillance radiograph during a scheduled postoperative clinic visit and qualified for subsequent analysis, whereas the remaining 97 were lost to follow-up after the index operation and/or did not receive a radiograph in the clinic. A flowsheet depicting patient selection is shown in Figure 1. Demographic information for the cohort is depicted in Table 1. One hundred forty-eight patients had femur fractures (53%), whereas 129 had tibial fractures (47%). Patients included in the study were significantly more likely to be male rather than female (67.5% male versus 32.5% female, *P* = 0.007). Tibial fractures were more likely to be open than femur fractures (34.1% vs. 17.6%, *P* = 0.003). There were no notable differences in age at the time of presentation, patient body mass index, or presence of fracture comminution between tibial and femoral fracture groups. The median patient follow-up was 23 weeks (Orthopaedic Trauma Association IQR - Inter Quartile Range [IQR], 9 to 30 weeks).

**Table 1:** Characteristics of the Cohort

		Missing	Overall	Femur	Tibia	*P* Value
N			277	148	129	
Age at presentation, mean (SD)		1	37.9 (18.9)	38.6 (20.7)	37.1 (16.7)	0.508
Sex, n (%)	F	0	90 (32.5)	59 (39.9)	31 (24.0)	0.007
	M		187 (67.5)	89 (60.1)	98 (76.0)	
BMI, mean (SD)		5	27.1 (6.6)	27.2 (6.8)	27.0 (6.4)	0.767
Open versus closed fracture, n (%)	Closed	0	207 (74.7)	122 (82.4)	85 (65.9)	0.003
	Open		70 (25.3)	26 (17.6)	44 (34.1)	
Comminution, n (%)	No	0	100 (36.1)	54 (36.5)	46 (35.7)	0.986
	Yes		177 (63.9)	94 (63.5)	83 (64.3)	
Screws, mean (SD)		10	4.2 (1.8)	3.7 (1.3)	4.8 (2.0)	<0.001

BMI = body mass index

A total of 617 radiographs were taken in 277 patients over the study period. Five hundred forty-six asymptomatic surveillance radiographs (88% of the total) and 71 clinically indicated radiographs were reviewed (12% of the total). Nine total radiographs were determined to have contributed to a change in the clinical plan (9/617 = 1.5% of total radiographs). Only one asymptomatic surveillance radiograph, taken at 14 weeks post-op in a patient with a femoral shaft fracture, resulted in a change in management (1/546 = 0.18% of asymptomatic radiographs). This radiograph demonstrated back out of the most distal interlocking screw of the intramedullary rod. The patient was informed and provided with return precautions should the implant become symptomatic. The patient later did become symptomatic, and screw removal proceeded 2 months after that visit. The remaining eight radiographs were performed with clinical correlations (eg, prominent metalwork or patient complaining of pain) and represent 11.3% of total clinically driven radiographs. The difference between the number of management-changing radiographs between asymptomatic versus clinically driven radiographs was significant (*P* < 0.001). The sensitivity analysis that was performed indicates that this difference is notable up until a threshold of five misclassified radiographs of the nine total (ie, five radiographs classified as being performed in a clinically indicated scenario should in fact have been defined as screening studies).

The details of radiographs resulting in altered management are represented in Table 2. No radiographs before 9 weeks post-op resulted in changes in management. For radiographs that lead to changes in management, five resulted in removal of migrated screws or metalwork and four in exchange nailing for nonunion. Based on these findings, the inclusion of asymptomatic screening radiographs in the first 14 weeks post-op for this cohort requires a number needed to screen of 357 radiographs to result in one radiograph that yields management-altering decision. Of the nine radiographs that changed management, five were in patients with tibia fractures, and four were in patients with femur fractures. There was no statistically significant difference between the number of patients who received management-changing radiographs between the femur and tibia groups (*P* = 0.81), nor was there a difference in the type of management-changing decision between tibia and femur fractures (screw removal versus surgical treatment for nonunion, *P* = 0.71). Patients who had management-changing radiographs were more likely to have had an open fracture rather than a closed injury pattern (*P* = 0.034).

**Table 2:** Characteristics of Management-Changing Radiographs

Week of Change	Fracture Site	Symptomatic or Asymptomatic	Finding	Outcome
9	Femur	Symptomatic	Prominent lateral screw	Planned removal of the screw
12	Tibia	Symptomatic	Migration of the screw	OR removal of the screw
14	Femur	Asymptomatic	Pull out of the most distal screw	Removal of the femoral screw
14	Tibia	Symptomatic	Migration of the distal screw near the knee	OR removal of metalwork
19	Tibia	Symptomatic	Nonunion	Planned amputation and prosthesis
22	Tibia	Symptomatic	Metalwork migration	Planned surgical removal
56	Tibia	Symptomatic	Nonunion	Planned exchange nailing
66	Femur	Symptomatic	Nonunion	Planned exchange nailing
73	Femur	Symptomatic	Nonunion	Planned exchange nailing

Historically, our institution performed an average of 161 tibial and femoral intramedullary fixations per year. Assuming a consistent proportion of tibial versus femoral fractures as found in our cohort (47% tibias and 53% femurs), the projected annual savings of eliminating all radiographs in the first 12 weeks after operation would be $300,958 using List Price values and $36,320 using Research Price values. Table 3 contains these results. All prices are in US dollars and are current for our institution as of March 2022.

**Table 3:** Cost Savings of Forgoing Surveillance Radiographs

Breakdown of Annual Lower Extremity Intramedullary Nail Incidence by Site		Imaging Study Cost		Total Annual List Price Savings by Eliminating Radiographs in the First 12 wk Post-op	
		List Price (Per Study)	Research Price (Per Study)	Based on List Price	Based on Research Price
Tibia	76/yr	$1042.95	$112.08	$79,264.20	$8518.08
Femur	85/yr	$837.82	$113.45	$71,214.70	$9643.25

List Price refers to price quoted to patients before insurance; Research Price reflects lowest possible charge to cover the cost of the study to institution. Estimate assumes that postoperative radiographs are taken at weeks 6, 12, and 24, as is our institution's paradigm.

## Discussion

In this study, the role of early postoperative radiographs in patients treated with intramedullary fixation for lower extremity long bone fractures was investigated using the radiologic information of a cohort of 277 patients. The research goal was to determine whether radiographs taken during these patients' visits made substantive changes to their overall treatment plans or whether they were ultimately unnecessary. Only nine patients in the cohort had radiographs that altered their treatment pathways, of which only one was conducted in an asymptomatic patient without a documented clinical correlate with their radiologic abnormality. Furthermore, no asymptomatic surveillance radiograph taken before 14 weeks post-op was found to alter patient treatment. These results suggest that early radiographs in clinically asymptomatic patients treated with lower extremity intramedullary nails are of little if any diagnostic yield. Such imaging represents both unnecessary costs on the healthcare system and clinical delays while waiting for imaging completion and radiation exposure for patients.

When looking at the radiographs that did change management, this study demonstrated two main outcomes: (1) radiographic evidence of migrated metalwork leading to removal and (2) radiographic evidence of nonunion that required surgical intervention (ie, exchange nailing). A study by Squyer et al^14^ shows that at the 3-month period, orthopaedic traumatologists are highly accurate in identifying patients at high risk for nonunion. Radiographs beyond the 3-month window are often clinically directed based on this prediction rather than surveillance for some asymptomatic irregularity. Our study shows congruent results in that asymptomatic surveillance radiographs in the first 12 weeks post-op frequently do not provide value or result in a change in management of nonunions.

To try and quantify the financial cost of poorly informative imaging studies, this investigation includes an estimate of the possible savings in eliminating surveillance radiographs in the first 12 weeks after operation. The estimate uses established face value pricing and does not include more complicated financial evaluations or review of specific hospital-related per-patient charges. Although these techniques may provide more specific information, they still often do not successfully capture the direct and indirect costs of a healthcare charge despite their complexity.^15,16^ Interpretation of cost savings is limited by the fact that only direct costs are considered, rather than also including associated fees and downstream effects of positive studies. In addition, our study does not take into account that radiographs are often used for purposes beyond monitoring for complications, including serving as a visual representation for patients of their healing. Further research should look into what proportion of patients themselves would forgo radiographs given that they are unlikely to discover complications versus how many would accept the financial and temporal cost in exchange for direct evidence of healing.

This study has several limitations. First, it is a single-center study and is not necessarily generalizable to other institutions with differing cadres of radiologists and surgeons. This is also a retrospective analysis of patient outcomes. A more complete study would prospectively look at patient outcomes in groups randomized to variable surveillance regimens, such as the work undertaken by Tufescu.^10^ However, their trial was relatively small, with only 39 patients randomized and 26 who received full follow-up. In addition, Tufescu did not focus solely on lower extremity diaphyseal fractures but rather any stable fracture fixation. This study took an all-comers approach regardless of fracture complexity and focused on a specific fixation method to provide more clinically relevant actionable data. As with any trauma study, patient follow-up is another limitation.^17^ Of the 374 patients included in this study, 97 did not follow-up in the clinic after their operation and did not receive any post-op imaging. Although a reality of the trauma cohort, this study's large cohort and pragmatic design hope to accurately reflect outcomes of patients with long bone fractures of the tibia and femur. In addition, it has both the investigators' experience and seen in the literature that patients with more complex injuries and persistent symptoms are more, rather than less, likely to return to the clinic for additional treatments.^18^ Finally, our classification of radiographs as asymptomatic versus clinically driven is a retrospective judgment based on information directly included by orthopaedic surgeons within their electronic visit note. It is possible that there is an element of clinical judgment in obtaining radiographs that is going undocumented and is therefore not captured by our approach. Future studies would include prospective surveillance regimen comparisons in which radiograph classification would occur before performing the imaging study. They should also stratify screening cohorts by whether they had an open or closed fracture, as this factor was found to notably affect the probability of a management-changing radiograph in our study.

Routine postoperative radiographs in patients with long bone fractures are ubiquitous and seemingly perpetuated by dogma and historical trends in orthopaedics. The results of this study suggest that surveillance radiographs in the first 12 weeks after tibial and femoral intramedullary fixation do not result in changes in management. A more clinically correlated practice pattern, whether it be by patient symptoms or examination findings, may result in fewer radiographic exposures for patients and reduce the overall healthcare cost.
